# Stage Ia squamous cell carcinoma as the malignant transformation of giant and unusual mature teratoma of the ovary in an elderly patient

**DOI:** 10.1186/s13048-022-01005-0

**Published:** 2022-06-04

**Authors:** Stefano Palomba, Tiziana Russo, Giuseppe Albonico, Marcello Tripodi

**Affiliations:** 1 Gynecology and Obstetrics, University Magna Graecia of Catanzaro, Catanzaro, Italy; 2 Casa di Cura “Caminiti”, Villa San Giovanni (RC), Italy; 3 Via A. Arabia 14, 87100 Cosenza, Italy; 4 Unit of Obstetrics and Gynecology, Grande Ospedale Metropolitano, Reggio Calabria, Italy; 5 Unit of Pathology, Grande Ospedale Metropolitano, Reggio Calabria, Italy

**Keywords:** Mature cystic teratomas, Ovarian cancer, Ovarian neoplasm, Ovary, Squamous cell carcinoma, Surgery

## Abstract

**Background:**

Mature cystic teratomas of the ovary (MCTO) are a type of germ cell tumor that may contain well-differentiated tissues developed from three germ cell layers and constitute about 20% of ovarian germ cell tumors. They are rare ovarian tumors with an annual incidence variable from 1.2 to 14.2 cases per 100,000 that occur mainly in women of reproductive age. They are frequently benign with a slow growth rate, even if they can undergo a malignant transformation in about 1–2% of cases.

**Case presentation:**

Here, we present the case of an elderly woman referred to gynecological first aid for acute abdominal pain and showing a giant and unusual MCTO at rapid growth with malignant transformation in squamous cell carcinoma (FIGO stage Ia). The patient underwent pelvic mass removal trough emergency longitudinal midline laparotomic incision with intraoperative frozen pathologic examination. A complete surgical staging during the first surgery was performed. After about 9 years of follow-up, she died of non-oncological reasons without recurrence.

**Conclusions:**

Present case highlights that CMTO with malignant transformation should always be suspected in elderly women in presence of pelvic mass at rapid growth, even if in absence of other clinical and ultrasonographic signs of malignancy. An intraoperative frozen pathologic examination may drive the best treatment.

## Background

Mature cystic teratomas of the ovary (MCTO) are a type of germ cell tumor that may contain welldifferentiated tissues developed from three germ cell layers, i.e., ectoderm, mesoderm, and endoderm [1–4]. They constitute about 20% of ovarian germ cell tumors [1–4]. The first case of MCTO was reported by Johannes Scultetus in 1659 after the autopsy of a young woman who died of an ovarian tumor and was described as a “dermoid cyst of the ovary”, while Rudolf Virchow introduced the term “teratoma” derived from the Greek word “teras,” meaning monster, in 1863 [5, 6]. The etiology, as well as the pathophysiology, of the MCTO, is not fully known; this is particularly true in consideration of their ability to differentiate into tissue cells and, in some cases, and they also develop into organ systems [7].

MCTOs are rare ovarian tumors with an annual incidence largely variable from 1.2 to 14.2 cases per 100,000 [8–11]. However, they are the most common benign tumor in women less than 45 years of age and predominant in women in their second and third decades of life [10]. They are also the most common ovarian mass in children [12]. They are always benign with a slow growth rate and, only in rare cases (about 2%), they can undergo malignant transformation [8, 9, 13]. Based on these considerations, the present study aimed to report the case of a giant MCTO with malignant transformation detected in an elderly woman.

## Case report

A 73-year-old (S.M. 24–08-39) woman presented at the gynecological first aid of the Grande Ospedale Metropolitano di Reggio Calabria (Italy) on the 22nd May 2012 referred with acute abdominopelvic pain. Her weight and height were, respectively, of 55 Kg and 155 cm. A detailed history with particular emphasis on gynecological data was taken. The woman was virgo, had a laparotomic appendectomy in childhood, had no pregnancy, and had been in menopause since the age of 50 years, receiving medication for hypertension. No other clinically relevant datum resulted from her medical and family history.

**Fig. 1:**
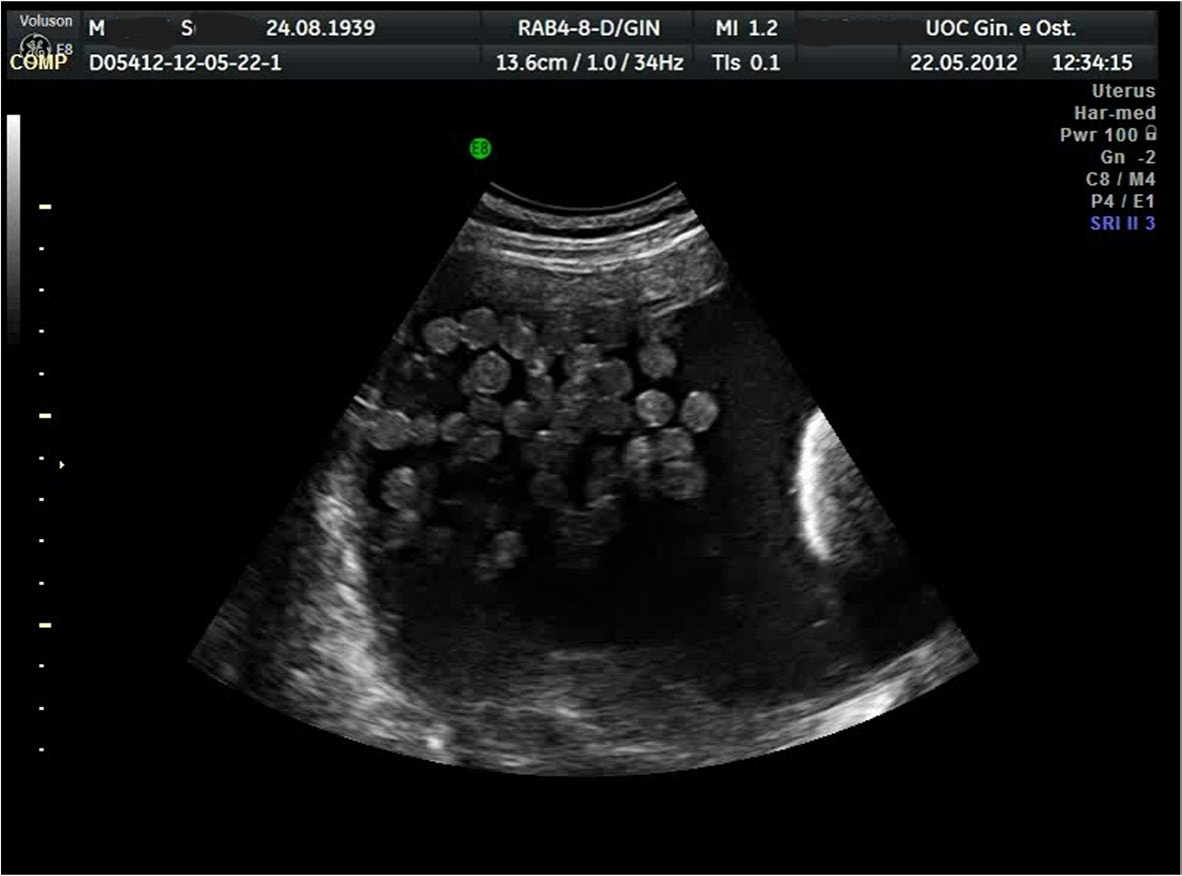
Initial ultrasound examination with evidence of pelvic mass consisting of uniloculated cyst of about 24 cm of maximum size with multiple small mobile hyperechoic internal lesions. An uneven area of about 2 cm located in the inferior cystic wall is also highlighted

**Fig. 2:**
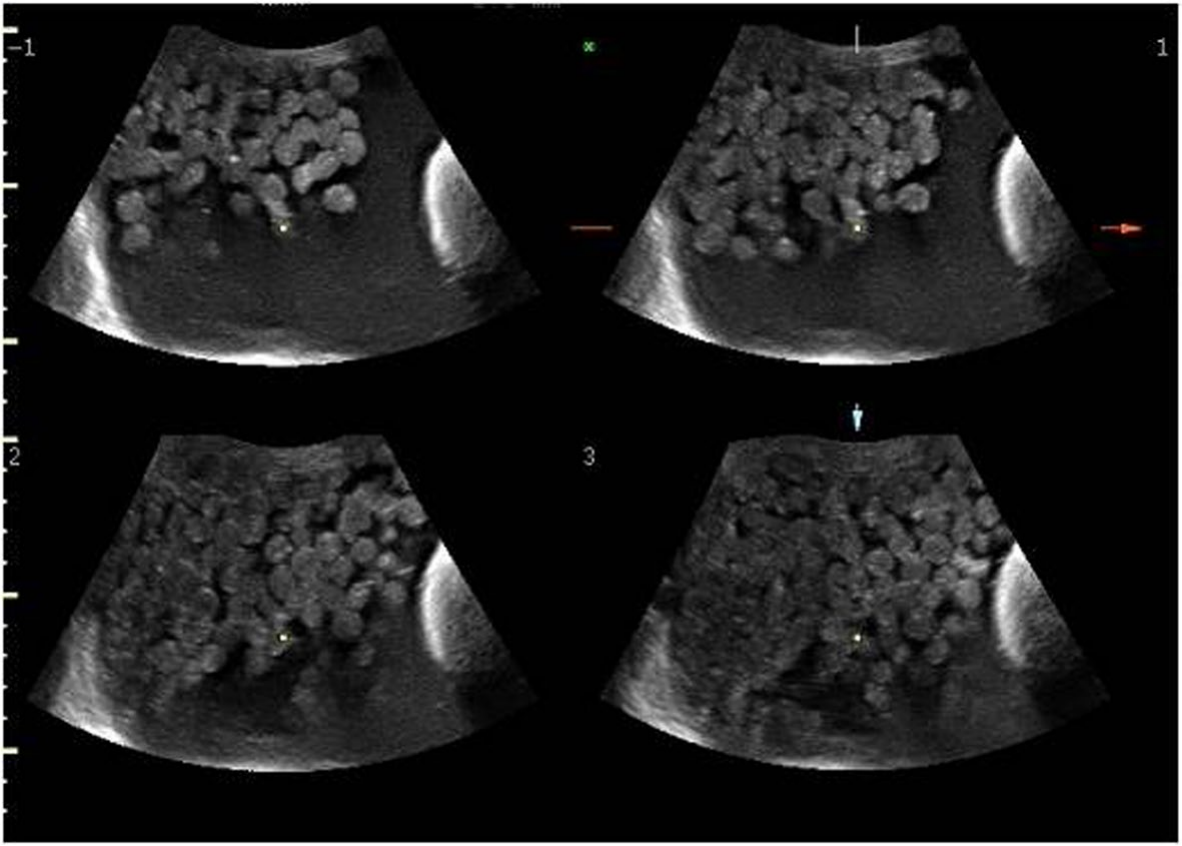
Ultrasound examination shows the extreme mobility of the endocystic formations to the movements impressed by the probe

**Fig. 3:**
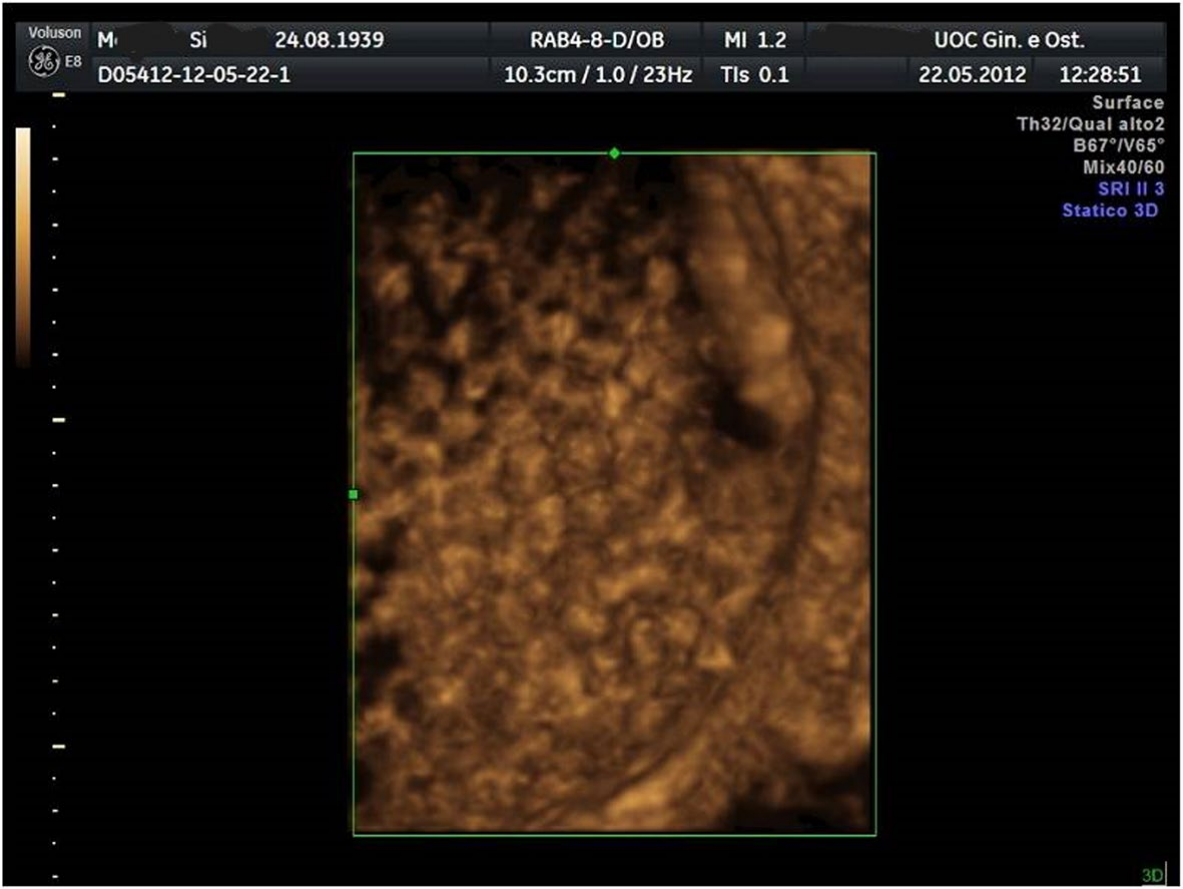
Static tridimensional (3D) ultrasound showing the “honeycomb” aspect

There was no history of weight loss or loss of appetite. Bowel and bladder habits were normal. The physical examination (including rectal examination) showed a palpable abdominopelvic mass with abdominal tenderness. The woman reported an increasing abdominal girth during the last 2 months. During the clinical examination, the patient had also nausea and vomiting. At admission, she was tachycardic (95 bpm) and tachypneic (16 breaths per minute). Initial transabdominal ultrasound examination was performed in an emergency. A pelvic mass consisting of a uniloculated cyst of about 24 cm of maximum size with multiple small mobile hyperechoic internal lesions was observed (Fig. 1). A solid and inhomogeneous structure of about 2 cm located in the inferior cystic wall was also observed (Fig. 1). No fluid or ascites was detected. An expert operator continued the ultrasound examination showing extreme mobility of the endocyst formations to the movements impressed by the probe (Fig. 2) and a “honeycomb” aspect was observed with the use of tridimensional ultrasound (3D-USG) (Fig. 3). No (typical/atypical) vascularization of the pelvic mass was detected at color Doppler velocimetry. Due to acute abdominal pain non-responsive to aggressive treatment with pain drugs, including a high dose of non-steroidal anti-inflammatory drugs (NSAD) (Toradol, ketorolac tromethamine, 2 vials of 10 mg/ml ev) plus (Contramal, tramadol hydrochloride, 1 vial of 100 mg/2 ml), it was decided for emergency surgery. The patient underwent a preoperative blood sample (for complete blood count, coagulation, and ovarian cancer markers) and electrocardiograms were transported to the operating room. Serum tumor markers, in particular, cancer antigen 125 (Ca 125), cancer antigen 19.9 (Ca 19.9), carcinoembryonic antigen (CEA), alphafetoprotein (AFP), human chorionic gonadotrophin (hCG), and lactate dehydrogenase (LDH) resulted in all normal ranges.

**Fig. 4:**
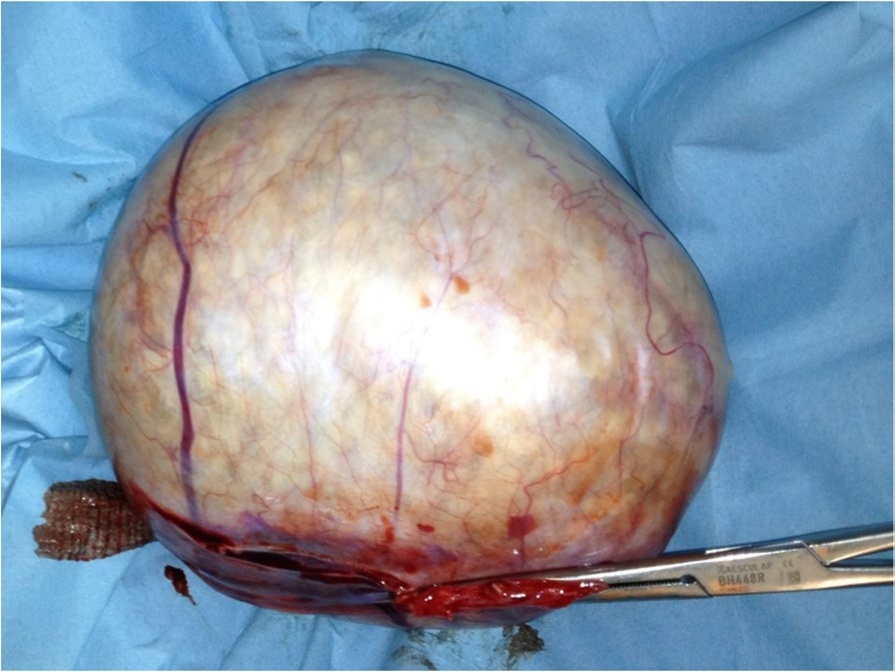
Macroscopic aspect of the left ovary after surgical excision. The dimensions measured after removal were 21 × 19 × 17 cm

**Fig. 5:**
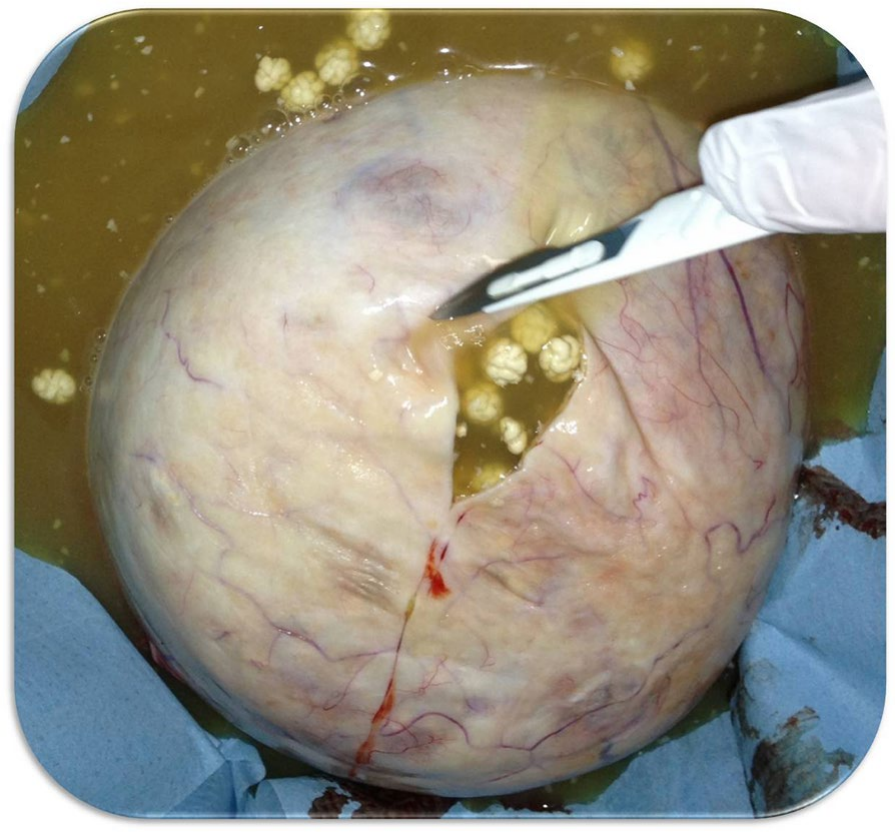
Macroscopic aspect of the mature cystic teratomas on opening. It is possible to observe citrine liquid with numerous and identical neoformations of adipose tissue of about 6mm each. A total of 184 neoformations were counted

**Fig. 6:**
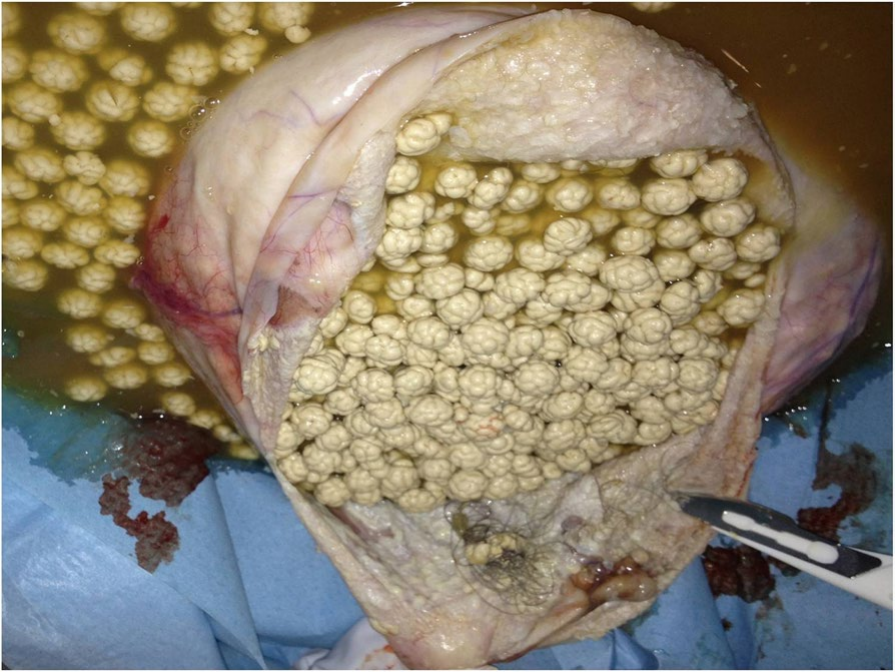
A calcific parietal implant with hair and an area of about one centimeter on the papillary surface was also detected. A biopsy was performed on this papillary structure for intra-operative histological examination

In consideration of the large mass and the little size of the abdomen, a longitudinal midline umbilicus-pubic skin incision was initially performed. Upon exploration of the abdomen, the pelvic mass was found to involve the entire left ovary, was mobile, and in partial torsion on its vascular peduncle. A left ovariectomy was initially performed without intra-abdominal spillage. The macroscopic aspect of the left ovary after surgical excision is shown in Fig. 4. The tumor was opened and inspected (Fig. 5). It had the macroscopic aspect of the MCTO and appeared to contain citrine liquid and numerous, small and identical neoformations. A calcific parietal implant with hair with a near area of about one centimeter of papillary tissue was also detected at the inspection of the internal capsule (Fig. 6). A biopsy was performed on this papillary structure for intra-operative histological examination. An intra-cystic area of squamous cell carcinoma was detected at the frozen section. Thus, a complete surgical staging for ovarian cancer, including total hysterectomy, bilateral adnexectomy, infracolic omentectomy, pelvic and para-aortic node dissection, and peritoneal washing, was performed. No local and/or distant suspected lesion was detected at the direct exploration of the pelvis, and the low and high abdomen. The patient was discharged from the hospital on the fifth day. No short- and long-term intraoperative or postoperative complications were found.

The definitive histological examination confirmed the presence of an MCTO measuring 21 × 19 × 17 cm with multiple neoformations of adipose tissue of about 6 mm each. A unique calcific parietal implant with hair was also observed. A papillary area of about one centimeter was described at the macroscopic examination. A total intra-cystic squamous cell carcinoma of moderate differentiation (grading 2) was also confirmed at microscopy. No local and/or distant metastasis was detected in all samples (uterus, surface of the left ovary, right ovary, left and right tubes, omentum, 8 right iliac nodes, 7 left iliac nodes, and 6 aortic nodes) sent for definitive microscopic examination. No cancer cells were also detected at the cytologic examination of the intracystic fluid and of the peritoneal washing. Thus, a Ia stage ovarian cancer according to the International Federation of Gynecology and Obstetrics (FIGO) was diagnosed.

After 30 days from hospital discharge, a computer tomography (CT) of the abdomen was performed confirming no disease. In consideration of the definitive histological report, the multidisciplinary group suggested only clinical follow-up. Follow-up visits were performed every 6 months for the first 3 years and subsequently yearly. The patient died of myocardial infarction on May 10, 2021.

## Discussion and conclusion

The current case regards an elderly patient referring to gynecological first aid for acute abdominal pain due to rapid growth of giant and unusual MCTO with malignant transformation in squamous cell carcinoma. The diagnosis was intraoperatively made and she underwent complete stadiation during the first surgery. She was followed up for about 9 years and died for non-oncological reasons.

The case may be interesting for several reasons. First of all, MCTOs are rare ovarian tumors with an annual incidence variable from 1.2 to 14.2 cases per 100,000 that occur mainly in young women and are rare in postmenopausal age [8–10]. Conversely, here we reported the case of CMTO diagnosed in an elderly woman. Although the clinician should always have a strong suspicion of malignancy in case of pelvic mass diagnosis in a woman of advanced age, such common benign neoformations should not be excluded from the differential diagnosis [14]. No clinical and ultrasound data of potential malignancy was observed at the exclusion of the rapid growth. We cannot precisely know how much before the CMTO was present but the patient referred an increase in the abdomen circumference during the previous 2 months suggesting a rapid growth rate. Conversely, MCTO are frequently benign with a growth rate lower than 2 cm/year [15, 16]. In addition, a malignant transformation in squamous cell carcinoma was detected at the histopathologic examination. Malignant transformation occurs in about 1–3% of cases of CMTO and squamous cell carcinoma is probably the most frequent [8–10]. Its incidence is 0.3% of cases [13]. Mucinous carcinomas, adenocarcinoma, undifferentiated carcinomas, type 2 papillary renal carcinoma, papillary thyroid carcinoma, malignant melanoma, choriocarcinoma, carcinoids, sarcomas, neuroectodermal tumors were also other types of malignant transformations [8–10, 13, 17].

The malignant transformation is a challenge for gynecologic oncologists since the preoperative detection is very difficult because the diagnostic accuracy of the imaging techniques is very low. The malignant transformation is usually detected post-operatively based on definitive histopathologic findings [13]. On the contrary, due to the clinical presentation in advanced age and as a giant adnexal mass suggesting a moderate risk for malignancy [18], the diagnosis was performed intraoperatively. In this regard, a longitudinal skin incision was planned. In fact, in consideration of the large mass and the little size of the abdomen (patient height was 155 cm), a laparoscopic approach was not considered sufficiently safe. The tumor was initially removed without intrabdominal spillage, the cyst was open and a biopsy from a papillary area was sent for frozen pathological examination. To date, this strategy was also recently considered valid in the case of risk factors for malignant transformation [13, 19, 20]. Clearly, it is possible to perform an intra-surgical frozen examination during emergency surgery only in referral Centers. After the intra-operative diagnosis of squamous cell carcinoma, a complete surgical staging including total hysterectomy with bilateral salpingo-oophorectomy, infra-colic omentectomy, pelvic and aortic lymphadenectomy, and peritoneal washing, was performed. The definitive histopathologic examination revealed no local and/or distant metastasis (FIGO stage Ia), and the patient was followed up without any postoperative treatment. After about 9 years of follow-up, the patients died for myocardial infarction without any recurrence. This favorable course of the disease, however, was associated with positive prognostic variables [13, 21, 22] notwithstanding no adjuvant chemotherapy. On the contrary, it is frequent to observe in the clinical practice the choice to avoid extensive complete staging (especially when the malignant transformation is detected postoperatively) and to start platinum-based chemotherapy in Stage Ia ovarian cancers [20].

An emergency longitudinal midline laparotomy was performed in our case study for acute abdominal pain. This was probably due to the partial torsion of the ovary. This is the most frequent complication of the CMTO with an incidence ranging from 3 to 21% most commonly occurring in CMTO with intermediate size and less frequent in case of CMTO of large dimensions [5, 6], as well as in our case. Commonly, a whirlpool sign with a twisted vascular pedicle is seen on color Doppler ultrasound in case of torsion. In our case, the color Doppler velocimetry was performed to detect potential pathological vascularization of the pelvic mass and no vascular flux was observed. Moreover, the dimensions of the mass make difficult the study of the vascular pedicle. On the other hand, trans-abdominal ultrasound was very accurate to study the abdominopelvic mass. Retrospectively, the typical ultrasound appearances of multiple floating (also known as “boba sign”) or non-floating balls (also known as “sack of marbles sign”) were clear [23–25]. Literature data [26, 27] have confirmed that ultrasound is accurate as magnetic resonance imaging (MRI) in the identification and characterization of CMTO. However, other imaging techniques were not performed in consideration of the acute abdominal pain requiring emergency laparotomy, even if CT of the abdomen and pelvis is believed to be the best modality for the preoperative evaluation of an adnexal mass [28].

Finally, our case was also unusual for the macroscopic appearance of the CMTO. The lesion here described was composed of ectodermal (cystic wall of skin with little small hair implants) and mesodermal tissue (little and identical balls of fat tissue) (Figs. 4 and 5). Ectodermal tissue (skin derivatives and neural tissue) is invariably present in CMTO and mesodermal tissue (fat, bone, cartilage, muscle) is present in over 90% of cases; endodermal tissue (e.g., gastrointestinal and bronchial epithelium, thyroid tissue) is also present in a high proportion of cases [1–4, 8–10].

The current case report confirmed that squamous-cell carcinoma in CMTO may be found, even if very rarely, in patients aged more than 50 years and with ovarian tumors more than 100 mm in size [10]. Moreover, in our case, the serum markers were in the normal range conversely to what was commonly suggested/observed [10]. Unfortunately, the serum human epididymis protein (HE4) assay, such as the squamous-cell carcinoma (SCC) antigen, was not measured because it was not available as a routine clinical practice in the Hospital during the period. Patients with FIGO stage Ia tumors have better survival than those with more advanced disease [10]. In this study, complete resection and staging were performed, no macroscopic/microscopic finding of metastasis was observed intraoperatively, post-surgery adjuvant chemotherapy was avoided and the patients had no recurrence during long-term follow-up. In this regard, an aggressive approach has recently been suggested to improve long-term survival [17].

In conclusion, our case highlights that CMTO may occur also in elderly women and that a malignant transformation should always be searched in presence of pelvic mass at rapid growth, even if in absence of other clinical and ultrasonographic signs of malignancy.

## Data Availability

The data used or analyzed are all in this published article.

## Abbreviations

3D-USG: Tridimensional ultrasound
AFP: Alpha-fetoprotein
Ca 125: Cancer antigen 125
Ca 19.9: Cancer antigen 19.9
CEA: Carcino-embryonic antigen
CT: Computer tomography
FIGO: International Federation of Gynecology and Obstetrics
hCG: Human chorionic gonadotropin
HE4: Human epididymis protein
LDH: Lactate dehydrogenase
MCTO: Mature cystic teratomas of the ovary
MRI: Magnetic resonance imaging
NSAD: Non-steroidal anti-inflammatory drugs
SCC: Squamous-cell carcinoma antigen
